# Photos Shared on Facebook in the Context of Safe Sleep Recommendations: Content Analysis of Images

**DOI:** 10.2196/54610

**Published:** 2024-04-23

**Authors:** Kelly Pretorius, Sookja Kang, Eunju Choi

**Affiliations:** 1 School of Nursing, Indiana University, Indianapolis, IN, United States; 2 School of Nursing, The University of Texas at Austin, Austin, TX, United States; 3 School of Nursing, MD Anderson Cancer Center, The University of Texas, Houston, TX, United States

**Keywords:** SUID, SIDS, parenting, safe sleep, photo analysis, pediatric, pediatrics, paediatric, paediatrics, infant, infants, infancy, baby, babies, neonate, neonates, neonatal, newborn, newborns, sleep, safety, death, mortality, social media, picture, pictures, photo, photos, photographs, image, images, Facebook, mother, mothers, parent, co-sleeping, sudden infant death, sudden unexpected infant death, adherence, parent education, parents' education, awareness

## Abstract

**Background:**

Sudden unexpected infant death (SUID) remains a leading cause of infant mortality; therefore, understanding parental practices of infant sleep at home is essential. Since social media analyses yield invaluable patient perspectives, understanding sleep practices in the context of safe sleep recommendations via a Facebook mothers’ group is instrumental for policy makers, health care providers, and researchers.

**Objective:**

This study aimed to identify photos shared by mothers discussing SUID and safe sleep online and assess their consistency with infant sleep guidelines per the American Academy of Pediatrics (AAP). We hypothesized the photos would not be consistent with guidelines based on prior research and increasing rates of accidental suffocation and strangulation in bed.

**Methods:**

Data were extracted from a Facebook mothers’ group in May 2019. After trialing various search terms, searching for the term “SIDS” on the selected Facebook group resulted in the most relevant discussions on SUID and safe sleep. The resulting data, including 20 posts and 912 comments among 512 mothers, were extracted and underwent qualitative descriptive content analysis. In completing the extraction and subsequent analysis, 24 shared personal photos were identified among the discussions. Of the photos, 14 pertained to the infant sleep environment. Photos of the infant sleep environment were then assessed for consistency with safe sleep guidelines per the AAP standards by 2 separate reviewers.

**Results:**

Of the shared photos relating to the infant sleep environment, 86% (12/14) were not consistent with AAP safe sleep guidelines. Specific inconsistencies included prone sleeping, foreign objects in the sleeping environment, and use of infant sleeping devices. Use of infant monitoring devices was also identified.

**Conclusions:**

This study is unique because the photos originated from the home setting, were in the context of SUID and safe sleep, and were obtained without researcher interference. Despite study limitations, the commonality of prone sleeping, foreign objects, and the use of both infant sleep and monitoring devices (ie, overall inconsistency regarding AAP safe sleep guidelines) sets the stage for future investigation regarding parental barriers to practicing safe infant sleep and has implications for policy makers, clinicians, and researchers.

## Introduction

In the United States, approximately 3500 infant deaths are attributed to the category of sudden unexpected infant death (SUID) on a yearly basis [1]. SUID includes death due to sudden infant death syndrome (SIDS), accidental suffocation and strangulation in bed, and ill-defined deaths [1]. SUID is the leading cause of unintentional, injury-related infant death in the United States [2] and is often related to unsafe infant sleep environments, including, but not limited to, prone sleeping, bed sharing, use of soft bedding, or unsafe sleep surfaces [3,4]. Recent characteristics of identified SUID deaths included that almost 60% of infants were sharing a sleep surface when they died and at least 76% had multiple unsafe sleep factors present [5].

The medical community has faced challenges in terminology associated with SUID [6], and the US Centers for Disease Control and Prevention recently updated reporting forms [7] to code such deaths more accurately. Confusion regarding SUID terminology also exists among parental groups [8]; for instance, parents often use the term “SIDS” when discussing SUID-related deaths. Progress related to the prevention of SUID has stalled since the Back to Sleep campaign in the 1990s [6,9]; therefore, further investigation into barriers to parental practices of safe sleep is warranted.

Social media is widely used among parenting groups and for health communication [10-12]. Mothers especially seek community and informational support—often found in online environments [8,13]. Given the breadth of information shared on social media, analyzing data from this source can identify concerns and practices of specific populations. Due to the stagnation in the prevention of SUID [1,2], we believed it would be helpful to complete a qualitative content analysis on Facebook and assess how mothers discuss SUID and safe sleep. In completing this analysis, we noted shared photos posted throughout the forum. Assessing infant sleep environments in the home setting is challenging [14]; thus, analyzing photos shared in an online community in the context of SUID and safe sleep discussions can yield invaluable insight into the reality of infant sleep environments. Understanding actual infant sleep environments in the home setting can also help in the development of research and prevention efforts regarding SUID.

This study therefore aimed to analyze photos shared among mothers engaged in discussions about SUID and safe sleep on a Facebook mothers’ group. Specifically, we were interested in the following question: If applicable, are the shared photos consistent with safe sleep guidelines as defined by the American Academy of Pediatrics (AAP) [1]?

## Methods

### Overview

Details regarding the extraction process and qualitative content analysis of the data have been published, and findings shared [8]; however, this will be briefly discussed here. Data were extracted from a Facebook mothers’ group in May 2019. This specific Facebook group was for women only, based in the southern United States, and had approximately 17,500 members. After trialing various options, “SIDS” was selected as the most effective term resulting in the most relevant conversations surrounding SUID and safe sleep. Thus, “SIDS” was entered in the search toolbar without additional filters applied. This search resulted in 20 posts and 912 comments from 512 mothers, all relevant to the topic of SUID and safe sleep. Once the data were identified, the posts and related conversations were transferred to an extraction spreadsheet and later analyzed via qualitative descriptive content analysis, as described by Sandelowski [15]. The descriptive analysis process was completed as per Miles et al [16] via Atlas.TI (ATLAS.ti Scientific Software Development GmbH) and was guided by the socioecological model of human development [17] and the uses and gratifications approach [18]. Inductive coding was completed so that codes emerged progressively, and all data were analyzed by 2 reviewers to ensure trustworthy findings and increase reliability [16].

During data extraction, photos shared among mothers were noted on the extraction spreadsheet. Two reviewers analyzed the photos to identify those related to the infant sleep environment. The identified photos related to the infant sleep environment were then assessed for consistency with safe sleep guidelines. The photos were analyzed based on 5 criteria derived from the AAP safe sleep guidelines (including risk factors and protective factors) that were current at the time of the analysis. These included (1) a supine sleep position, (2) no bed sharing, (3) the absence of soft bedding (crib bumpers, positioners), (4) a safe sleep surface, and (5) pacifier use [3]. If there was a discrepancy in assessing the safety of the environment, this was discussed among the members until a consensus was reached.

### Ethical Considerations

This study was submitted for review by the institutional review board (IRB) at the University of Texas at Austin and deemed exempt as the study did not meet the criteria for human subjects research. IRB review and oversight was not required because the activities involved obtaining information from publicly available data sets. Despite the exempt status, any personal or identifying information was removed from the data set to maintain confidentiality. Thus, privacy and confidentiality were maintained throughout the data collection and analysis process. Additionally, in discussing the findings, we have chosen to share minimal details regarding specific posts and will not share the actual photos included in this analysis.

## Results

### Sleep Environment Assessment

Among the data extracted from the Facebook group, 24 personal photos were identified. Of these photos, 14 were related to the infant sleep environment, 10 of which included sleeping infants. The remaining photos, which were not included, depicted infants and toddlers that were awake, personal photos of infant monitoring devices, and screenshots of personal monitoring device results via applications originating from monitoring devices.

After review, 8 of the 10 photos of infants sleeping were classified as being inconsistent with AAP safe sleep guidelines. Reasons for being deemed inconsistent included prone sleeping, the use of sleeping devices, and foreign objects in the sleeping environment (blankets, stuffed animals, crib bumpers). Four of the photos depicted infant sleeping environments but did not have the infant present. Despite the absence of the actual infant, the shared photos depicting sleeping environments were all classified as being inconsistent with AAP safe sleep guidelines after review. Examples of these environments included foreign objects in the sleeping environment (blankets, stuffed animals, diapers, wipes). In evaluating the photos shared among this specific Facebook group, it is important to consider the context in which the photos were initially shared.

### Context of Shared Photos

To better understand the mothers’ intentions in sharing photos, some of the scenarios will be discussed in more detail. However, it is worth noting that significant effort was made to maintain the anonymity of the Facebook members and actual photos are not available for viewing.

Most of the photos shared among members were “for attention” or to discuss infant sleeping habits rather than to clarify whether the sleeping environment was safe. For example, a mother was considering using crib bumpers and asked the group for advice since her infant slept with his head touching the side of the crib. In posting her question, she shared a photo of her infant in the crib, in which there were multiple foreign objects present and the infant was sleeping prone. This resulted in other shared photos among the mothers. For instance, a mother commented, “This is how my son sleeps! We have used crib bumpers with all 3 of my kids” and included a photo of her infant sleeping prone in the crib with crib bumpers present. She also added there were “cute” crib bumpers available via Amazon. A different mother stated, “I’ve used a bumper with every one of mine” and shared a photo of her infant, supine with a stuffed animal and crib bumpers present in the crib.

When another mother asked how many parents went against pediatrician recommendations for infants sleeping on their backs, she posted a photo of her infant sleeping prone on a blanket with the caption “Picture of my LO for attention!!” In response to this post, another mother shared a photo of her infant sleeping prone in their crib from that morning and commented “Do what works for you!” Within the same concept of prone sleeping, another post inquired about mothers who have infants that “tummy” sleep and shared a photo of her infant in her lap, “just because.”

One mother asked the group for advice for “any product similar” to the Owlet baby monitor (Owlet Baby Care Inc), as she was looking for a solution to “always getting up to check that they’re still breathing.” In response to this question, a different mother commented, “DO IT!!” Along with this comment, this mother shared a photo of her infant, aged 4 months, sleeping in a Rock n Play (Fisher-Price Inc) with a neck bandana and an Owlet.

The selected Facebook group also discussed transitioning infants to different sleep settings, such as from a bassinet to a crib. This topic resulted in shared personal photos of various sleeping environments. For example, one mother demonstrated her transition to a crib via a pack and play pictured with netting cut out and multiple foreign objects present. Another shared photo depicted a bassinet in which the mother emphasized the importance of keeping her essentials, “diapers and stuff,” in the bassinet in order to be successful with nighttime diaper changes.

It is worth noting that one of the personal photos of an infant sleeping (classified as being consistent with AAP safe sleep guidelines) was an infant lying on their back, with the glow of a baby monitoring device clearly visible through their onesie. Another photo of an infant that was awake (also classified as consistent with safe sleep guidelines based on the visualized sleep environment) included text referencing that the infant slept in a bassinet, “in the middle of our bed,” thus implying an unsafe sleep environment in actuality.

Only one photo in this analysis was posted with the mother’s intent to ensure a safe sleep environment. This mother sought confirmation that her infant was sleeping “appropriately” and shared a personal photo. This was one of the few environments consistent with safe sleep guidelines among the sample, as there were no foreign objects in the crib. In summary, among the main posts and associated comments, 14 personally shared photos were related to the infant sleep environment, and 86% (12/14) were inconsistent with AAP safe sleep guidelines.

## Discussion

### Unsafe Infant Sleep Practices

These specific data are unique in that the users openly shared their photos in a forum, a Facebook mothers’ group, without researcher interference. The photos were also shared within the context of discussing SUID and safe sleep. It is therefore interesting that most of the shared photos demonstrated sleep environments inconsistent with AAP safe sleep guidelines.

Our findings are concordant with prior analyses of actual infant sleep practices [14], although our analysis involved photos and assessed the home setting rather than an artificial environment. Prior research on this topic has identified unsafe sleep environments depicted on Instagram [19], in stock photographs [20], in magazines [21], in crib marketing [22], and among websites resulting from a Google search [23].

At the time of the study, AAP guidelines were clear regarding use of a firm sleep surface and restriction of objects in the crib, yet guidelines were not clear regarding infant monitoring devices. Thus, repeating this study after device recalls [24-27] and subsequent federal regulations on the use of infant sleep devices [28] might be worthwhile.

### Limitations

This study is not without limitations. The number of photos analyzed is small and does not necessarily represent the general population. Furthermore, the photos analyzed represent snapshots and may not fully capture typical sleeping conditions. Additionally, there is concern for potential bias in what photos were shared among the members and in what context. For example, a member may have shared a photo if they were unsure about the safety of the environment; therefore, this study might overestimate environments inconsistent with AAP safe sleep guidelines. However, when assessing the context of the shared photos, only one photo was shared with the mother’s intention of inquiring about the safety of the sleep environment.

Results from this photo analysis do not necessarily represent all parents, especially those who do not use Facebook or engage in groups on Facebook. Demographic information regarding the mothers was also not obtained, further limiting generalizability of the findings and resulting in an inability to verify the accuracy of the information shared by mothers.

Despite such limitations, visualizing the actual sleep environment in a naturalistic setting provides helpful insight into true parental practices of infant sleep and has implications for future research and practice.

### Conclusions

The commonality of sleep environments inconsistent with AAP safe sleep guidelines should be considered by policy makers, health care professionals, and researchers when aiming to prevent the occurrence of SUID. The practice of infant sleep is complex [8], and despite the known risks of bed-sharing, parents are often motivated to use infant sleep practices inconsistent with AAP guidelines [29]. Additionally, infant sleep is a controversial parenting topic, and while bed-sharing is discouraged in the United States, this practice is commonplace worldwide [30]. While much research focuses on knowledge-based interventions regarding infant sleep practices, we suggest a shift to focus on supporting parents so they can create a sleep environment consistent with AAP guidelines.

This shift involves broad policy changes, such as paid parental leave and financial assistance so families can afford to live in a home with a sufficient number of bedrooms or can purchase safe environments for their infant to sleep (eg, a pack and play or crib). Other suggested policy changes include extending health care coverage for the birthing person up to a year postpartum to ensure adequate care and social services for the mother-infant dyad. Additionally, continued federal regulations are needed to safeguard families. Research has identified widespread use [8] and popularity of infant sleep and monitoring devices among families [31]; however, despite deaths attributed to the use of these devices, their regulation [24-27] has lagged. For instance, the Safe Sleep for Babies Act of 2021 [28], which bans inclined sleepers and crib bumpers, only passed in 2022 despite documentation that such devices have contributed to infant deaths since the 1990s [32].

Health care organizations and personnel should aim to emulate a comfortable environment where parents can engage in open discussions about their infant sleeping practices. Parents seek support from social media [8,11] because it is often a place of acceptance; health care professionals should aspire to be another source of support while providing anticipatory guidance regarding infant sleep. Additionally, health care providers should approach this conversation with the intent to empower parents to create safe sleep environments while having honest conversations about barriers to these practices. Health care personnel should also aim to address infant sleep environments when counseling parents—beginning at pregnancy and throughout the infant’s first year of life.

Lastly, since SUID remains the leading cause of unintentional, injury-related infant death in the United States [2], future research should prioritize investigating parental barriers to creating sleep environments consistent with AAP guidelines. Efforts should be made to find new ways to support parents, rather than focusing on knowledge-based interventions. In conclusion, this study highlights the critical need for policy makers, health care professionals, and researchers to engage in the prevention of deaths attributed to SUID by supporting families at the interpersonal, community, and system levels.

## Abbreviations

AAP: American Academy of Pediatrics
IRB: institutional review board
SIDS: sudden infant death syndrome
SUID: sudden unexpected infant death

## References

[1] Moon RY, Carlin RF, Hand I (2022). Evidence base for 2022 updated recommendations for a safe infant sleeping environment to reduce the risk of sleep-related infant deaths. Pediatrics.

[2] (2020). 10 leading causes of injury deaths by age group. US Centers for Disease Control and Prevention.

[3] Carlin RF, Moon RY (2017). Risk factors, protective factors, and current recommendations to reduce sudden infant death syndrome: a review. JAMA Pediatr.

[4] Moon RY, Hauck FR (2015). Hazardous bedding in infants’ sleep environment is still common and a cause for concern. Pediatrics.

[5] Erck Lambert AB, Shapiro-Mendoza CK, Parks SE, Cottengim C, Faulkner M, Hauck FR (2024). Characteristics of sudden unexpected infant deaths on shared and nonshared sleep surfaces. Pediatrics.

[6] Hauck FR, Tanabe KO (2008). International trends in sudden infant death syndrome and other sudden unexpected deaths in infancy: need for better diagnostic standardization. Pediatrics.

[7] SUIDI reporting form. US Centers for Disease Control and Prevention.

[8] Pretorius K, Choi E, Kang S, Mackert M (2020). Sudden infant death syndrome on Facebook: qualitative descriptive content analysis to guide prevention efforts. J Med Internet Res.

[9] Erck Lambert AB, Parks SE, Shapiro-Mendoza CK (2018). National and state trends in sudden unexpected infant death: 1990-2015. Pediatrics.

[10] Moon RY, Mathews A, Oden R, Carlin R (2019). Mothers’ perceptions of the internet and social media as sources of parenting and health information: qualitative study. J Med Internet Res.

[11] Pretorius K, Johnson KE, Rew L (2019). An integrative review: understanding parental use of social media to influence infant and child health. Matern Child Health J.

[12] Huo J, Desai R, Hong YR, Turner K, Mainous AG, Bian J (2019). Use of social media in health communication: findings from the Health Information National Trends Survey 2013, 2014, and 2017. Cancer Control.

[13] Duggan MLA, Lanhart A, Lampe C, Ellison NB (2015). Parents and social media: mothers are especially likely to give and receive support on social media. Pew Research Center.

[14] Batra EK, Teti DM, Schaefer EW, Neumann BA, Meek EA, Paul IM (2016). Nocturnal video assessment of infant sleep environments. Pediatrics.

[15] Sandelowski M (2000). Whatever happened to qualitative description?. Res Nurs Health.

[16] Miles MH, Michael A, Saldana J (2014). Qualitative Data Analysis: A Methods Sourcebook.

[17] Bronfenbrenner U (1979). The Ecology of Human Development.

[18] Ruggiero TE (2000). Uses and gratifications theory in the 21st century. Mass Commun Soc.

[19] Kanits F, L’Hoir MP, Boere-Boonekamp MM, Engelberts AC, Feskens EJM (2023). #sleepingbaby on Instagram: nonadherence of images to safe sleeping advice and implications for prevention of sudden unexpected death in infancy. PLoS One.

[20] Goodstein MH, Lagon E, Bell T, Joyner BL, Moon RY (2018). Stock photographs do not comply with infant safe sleep guidelines. Clin Pediatr (Phila).

[21] Joyner BL, Gill-Bailey C, Moon RY (2009). Infant sleep environments depicted in magazines targeted to women of childbearing age. Pediatrics.

[22] Kreth M, Shikany T, Lenker C, Troxler RB (2017). Safe sleep guideline adherence in nationwide marketing of infant cribs and products. Pediatrics.

[23] Chung M, Oden RP, Joyner BL, Sims A, Moon RY (2012). Safe infant sleep recommendations on the Internet: let’s Google it. J Pediatr.

[24] Trumka R (2022). Dockatot Deluxe+ is unsafe for sleep; CPSC issues notice of violation. US Consumer Product Safety Commission.

[25] (2023). Fisher-Price reannounces recall of 47 million Rock ‘N Play sleepers; at least eight deaths occurred after recall. US Consumer Product Safety Commission.

[26] (2021). Warning letter: Owlet Baby Care, Inc. US Food & Drug Administration, Center for Devices and Radiological Health.

[27] (2021). Final rule: safety standard for infant sleep products. Federal Register.

[28] (2022). Hr3182 - Safe Sleep for Babies Act of 2021. US Congress.

[29] Chianese J, Ploof D, Trovato C, Chang JC (2009). Inner-city caregivers’ perspectives on bed sharing with their infants. Acad Pediatr.

[30] Mileva-Seitz VR, Bakermans-Kranenburg MJ, Battaini C, Luijk M (2017). Parent-child bed-sharing: the good, the bad, and the burden of evidence. Sleep Med Rev.

[31] Pretorius KA, Mackert M, Wilcox GB (2018). Sudden infant death syndrome and safe sleep on Twitter: analysis of influences and themes to guide health promotion efforts. JMIR Pediatr Parent.

[32] (2020). Proposed rule: safety standard for crib bumpers/liners under the Danny Keysar Child Product Safety Notification Act. US Consumer Product Safety Commission.

